# Does Cognitive Stimulation Therapy show similar efficacy in individuals with mild-to-moderate dementia from varying etiologies? An examination comparing its effectiveness in Alzheimer's disease and vascular dementia

**DOI:** 10.1016/j.ijchp.2024.100510

**Published:** 2024-10-18

**Authors:** Federica Piras, Elena Carbone, Riccardo Domenicucci, Enrico Sella, Erika Borella

**Affiliations:** a Neuropsychiatry Laboratory, Clinical Neuroscience and Neurorehabilitation Department, IRCCS Fondazione Santa Lucia, Via Ardeatina 306-354-00179, Rome, Italy; b Department of General Psychology, University of Padova, via Venezia 8 -35131, Padova, Italy

**Keywords:** Evidence-based psychosocial interventions, Cognitive stimulation therapy, Cognitive-neuropsychiatric profiles in dementia, Vascular dementia, Depression, Quality of life

## Abstract

**Objective:**

The effectiveness of the Cognitive Stimulation Therapy (CST) is well-documented. Nevertheless, the question of whether specific subgroups of individuals with dementia are more or less likely to benefit from this cognitive stimulation intervention remains unaddressed. Here, we directly compared the effectiveness of the Italian CST (CST-IT), delivered in a previous multicenter controlled clinical trial, across two distinct cohorts of individuals clinically diagnosed with Alzheimer's Disease (AD, *N* = 30) and vascular dementia (VaD, *N* = 27) in the mild-to-moderate stage.

**Method:**

The impact of dementia subtype (AD vs VaD) on immediate (at intervention completion) benefits of CST-IT in general cognitive functioning, communicative abilities, mood, behavior and perceived quality of life was evaluated through linear mixed effects models. The frequency and severity of neuropsychiatric symptoms at baseline was inserted as a covariate due to the different behavioral profile in the two groups. Exploratory analyses also investigated the potential differential effect of dementia subtype on long-term benefits (three months after intervention).

**Results:**

The CST-IT determined comparable immediate, clinically significant improvements in general cognition and communicative abilities. Dementia subtype influenced short-term benefits in depressive symptoms (with a greater decrease in AD patients) and quality of life (no significant impact in AD, and a small improvement in VaD). Such effects depended on diagnosis-related differences in neuropsychiatric symptoms. At long-term, benefits persisted in general cognition (though depending on the outcome considered). Improvements in narratives were seen in VaD, whereas communicative abilities in AD returned to baseline. Post-intervention gains in depressive symptoms persisted in AD, but not in VaD, although benefits in quality of life remained stable in the latter.

**Conclusions:**

Different mechanisms of neuropsychological change after CST-IT were hypothesized for the different forms of dementia, particularly with respect to crucial outcomes such as language, mood and quality of life, with implications toward the delivery of such psychosocial intervention in clinical contexts.

## Introduction

The effectiveness of Cognitive Stimulation Therapy (CST) (Spector et al., 2003, 2006) as a psychosocial intervention aimed at augmenting general and specific cognitive functions in individuals with mild-to-moderate dementia has been firmly substantiated (Desai et al., 2024; Woods et al., 2023). Likewise, the fact that slight improvements in quality of life, mood, activities of daily living as well as decrease of neuropsychiatric symptoms -an important addition to cognitive benefits- may be associated with cognitive stimulation, has been corroborated by an increasing number of studies (Woods et al., 2023).

To date, group-based CST has been recommended by the UK National Institute for Health and Care Excellence guidelines (National Collaborating Centre for Mental Health (UK), 2007) and Alzheimer's Disease International (Prince, Comas-Herrera, Knapp, Guerchet, & Karagiannidou, 2016), successfully filling the existing gaps between limited pharmacological treatments and the urgent needs of people living with dementia. Since its inception, CST has been translated into at least eight different languages and implemented in 24 nations, demonstrating its effectiveness and widespread applicability (Alvares Pereira, Sousa, & Nunes, 2022; Capotosto et al., 2017; Wong, Yek, Zhang, Lum, & Spector, 2018). Rooted into reality orientation therapy, reminiscence therapy, multisensory stimulation and implicit learning, and taking advantage of the positive aspects of such therapeutic techniques and principles (Spector, Orrell, Davies, & Woods, 2000), the CST ensures that people with dementia are stimulated in a sensitive, respectful, and person-oriented way. Through a series of engaging activities carried out in small groups that stimulate thinking, memory and orientation over 14 structured thematic cognitive stimulation sessions, this evidence-grounded program combines a cognition-based approach with psychosocial and relational features and is aimed at enhancing cognitive-social functioning (Clare & Woods, 2003).

While several studies have shown CST efficacy in counteracting cognitive decline among individuals with dementia when compared to standard care (Chen, 2022) or active group interventions (Carbone et al., 2021; Lobbia et al., 2019), recent network meta-analyses (Luo et al., 2023; Wang et al., 2020) have examined its comparative effectiveness against other non-pharmacological treatments. These analyses have identified CST as the most beneficial intervention, along with exercise therapy, for individuals with dementia (Luo et al., 2023), while the program exhibits the highest likelihood of being the optimal treatment also for people with mild cognitive impairment (Wang et al., 2020).

Nevertheless, previous studies examined (and compared) CST effectiveness in mixed samples of patients affected by Alzheimer's disease (AD) and related dementias (ADRD) and to date, no studies have specifically addressed the potential differences in program effectiveness based on diagnosis. Although potentially confounded by a medication effect, as symptomatic treatments are not available for all dementia forms (Woods et al., 2023), the matter at hand is significant especially considering the established variations in the cognitive and behavioral characteristics among clinically identified subtypes of dementia, which are indicative of the underlying neuropathology (Devanand, Lee, Huey, & Goldberg, 2022).

Based on these assumptions, and as previously suggested by Woods et al. (2023), further work is needed to determine if certain individuals with dementia, or specific subgroups, are more or less likely to benefit from cognitive stimulation interventions. Therefore, we directly compared the effectiveness of the Italian CST adaptation (CST-IT) (Capotosto et al., 2017) delivered in a previous single-blind (assessor-blinded), multicenter, controlled clinical trial (Carbone et al., 2021) across two distinct cohorts of individuals clinically diagnosed with AD and vascular dementia (VaD) in the mild-to-moderate stage. Indeed, the diagnostic criteria for VaD have been clearly dissociated from those of AD (Román et al., 1993) and in the last decade several new criteria have been developed to broaden the concept of VaD, as to better reflect the full range of cognitive alterations resulting from vascular factors (Chui et al., 2000). The cognitive profile of VaD is characterized by a more pronounced dysexecutive syndrome and greater deficits in processing speed than non-vascular cognitive disorders (Vasquez & Zakzanis, 2015). Yet, a significant intersection exists in the impairments noted in both AD and VaD, underscoring the intricate nature of VaD pathophysiology, which incorporates elements of both neurodegeneration and vascular mechanisms. Also considering neuropsychiatric symptoms, which frequently occur in AD and ADRD, distinctions can be observed between these two diagnostic entities. Specifically, apathy, anxiety and depression are frequently observable in individuals along the ADRD continuum, while the prevalence of psychosis, characterized by delusions or hallucinations, tends to be lower in individuals with VaD, with rates of up to 45% in AD patients (Devanand et al., 2022). Agitation, irritability, aberrant motor behavior, and psychotic or manic syndromes, including disinhibition and euphoria, exhibit an escalated occurrence as the severity of AD increases (Spalletta et al., 2010) while apathy, sleep disturbances, anxiety, and depression increase as the disease progresses in VaD patients (Kazui et al., 2016).

Considering the significant relationship between neuropsychiatric symptoms, global cognition and cognitive domains, such as the link between apathy and general cognitive decline, as well as between delusions and impaired executive function (Sabates et al., 2024), it is anticipated that increased affective and behavioral symptomatology in people with dementia is associated with worse cognitive performance. This would also imply a distinct relationship between neuropsychiatric symptoms and cognitive profiles in AD and VaD. The interwoven nature of this relationship may exhibit varying interactions with the effectiveness of the CST-IT program, also considering both the direct and indirect influences of the cognitive/neuropsychiatric profile on important outcome indicators such as quality of life (Woods, Thorgrimsen, Spector, Royan, & Orrell, 2006) and mood (Chen, 2022). Although the effect of the CST program on neuropsychiatric and affective symptoms is debated (Chen, 2022; Lobbia et al., 2019), we previously demonstrated (Capotosto et al., 2017) that CST-IT determined a reduction in depressive symptomatology (along with better general cognition, language abilities and perceived quality of life) in a mixed sample of dementia patients. Such benefits were maintained in the long-term (3 months after intervention completion), while symptoms of depression exhibited a progressive rise in the active control group, especially among individuals with pronounced cognitive deficits at baseline (Carbone, Piras, Pastore, & Borella, 2022), thus confirming once again, the complex interplay between cognitive functioning and depressive manifestations. Contrariwise, when the effect of CST-IT was evaluated in a sample of VaD patients only (Piras et al., 2017), general cognitive functioning improved at program completion (with a trend toward significance for working memory abilities), but no positive impact was observable on mood and behavior, suggesting that different mechanisms of change are at play when patients with a prominent vascular contribution to dementia are considered.

Therefore, the present comparative study directly investigated the potential effect of dementia subtype on short- and long-term benefits derived from the CST-IT program in general cognitive functioning and specific cognitive domains (i.e., language skills), mood and behavior and perceived quality of life. Based on our previous findings (Capotosto et al., 2017; Carbone et al., 2021, 2022; Piras et al., 2017) we expected short- and long-term improvements in general cognitive functioning, language skills and quality of life in both groups, while significant changes in mood and behavior were anticipated in the AD sample only (Piras et al., 2017). In line with prevalence studies (Devanand et al., 2022) we also expected more frequent and severe neuropsychiatric symptoms in the AD sample, a significant association between VaD and affective symptomatology (according to diagnostic criteria) (Román, 2004) and a potential interaction between neuropsychiatric symptoms, cognition and quality of life (Sabates et al., 2024). Although the investigation of long-term benefits was purely exploratory (considering the different attrition rate in the two samples), given the irreversible nature of the deficits and decline in autonomy linked to AD in contrast to the response to interventions in VaD, it was expected that individuals with a predominantly vascular-related cognitive impairment would display a differential preservation of improvement. Our findings might indeed shed light on the processes of change facilitated by the CST program in this cohort of patients affected by both vascular and neurodegenerative mechanisms.

## Methods

### Participants

Data on people with dementia involved in the CST-IT in a previous single-blind (assessor-blinded), multicenter, controlled clinical trial (Carbone et al., 2021) were examined. In particular, participants diagnosed with Alzheimer's disease (AD) and with vascular dementia (VaD) of the treatment group were selected. We included a total of 58 participants with dementia assigned to the CST treatment. A participant with VaD was excluded due to incorrect enrollment. Therefore, the final sample comprised 57 participants, 30 of them diagnosed with Alzheimer's disease (AD) and 27 with vascular dementia (VaD). The two groups did not differ in terms of age, gender, education and Clinical Dementia Rating scores (see Table 1).

**Table 1:** Descriptive statistics of socio-demographic characteristics and measures of interest by group (AD and VaD) and assessment session (pre-test and post-test) and results of groups comparisons at baseline.

	*Baseline differences*	AD (*N* = 30; 19 females)										VaD (*N* = 27; 22 females)									
		*M*			*SD*				*Min-Max*			*M*			*SD*				*Min-Max*		
Age	*U* = 283; *p* = .052	80.07			6.21				68–90			82.33			8.97				55–92		
Education	*U* = 301; *p* = .08	7.03			3.03				1–13			5.67			2.73				1–13		
CDR	*U* = 316; *p* = .48	1.39			0.50				1–2			1.30			0.50				0.5–2		
	*Baseline differences*	Pre-test					Post-test					Pre-test					Post-test				
		*N*	*M*	*SD*		*Min-Max*	*N*	*M*		*SD*	*Min-Max*	*N*	*M*	*SD*		*Min-Max*	*N*	*M*		*SD*	*Min-Max*
MMSE	*U* = 391; *p* = .82	30	19.64	3.59		12–24	30	20.41		4.08	13–27	27	19.63	3.43		14–24	27	20.78		4.46	9–28
ADAS-Cog	*U* = 331; *p* = .91	26	28.99	12.79		15–65	26	24.94		13.63	10–66	26	26.84	8.97		14–50	26	24.71		9.17	13–51
NLT	t(55) = 1.67; *p* = .10	30	12.17	4.91		2–23	30	16.13		7.53	5–33	27	10.07	4.50		1–17	27	13.37		5.20	3–24
CSDD	*U* = 305; *p* = .11	30	7.40	5.12		0–20	30	4.17		3.44	0–11	27	5.41	4.99		0–16	27	3.70		3.90	0–15
NPI	***U*=225; *p* = .004**	30	16.30	13.05		0–46	30	11.37		11	0–45	27	7.81	11.76		0–49	27	6.41		8.04	0–34
QoL-AD	*U* = 288; *p* = .06	30	31.73	8.51		6–43	30	31.63		7.51	6–42	27	27.63	8.97		7–42	27	30.15		7.54	13–44

**Note**: *AD = Alzheimer's Disease; VaD = Vascular Dementia; CDR = Clinical Dementia Rating; MMSE = Mini-Mental State Examination; ADAS-Cog = Alzheimer's Disease Assessment Scale – Cognitive Subscale; NLT = Narrative Language Test; CSDD = Cornell Scale for Depression in Dementia; NPI = Neuropsychiatric Inventory; QoL-AD = Quality of Life in Alzheimer's Disease Scale. U = Mann-Whitney U test; t = independent sample t-test. Significant results in bold.*

The study was approved by the local research ethics committee for psychological research and the experimental procedure complied with the principles of the Declaration of Helsinki.

### Measures

#### General cognitive functioning

Mini-Mental State Examination (MMSE) (Folstein, Folstein, & McHugh, 1975). It is the most widespread cognitive screening test for dementia (Di Pucchio et al., 2018) comprising items for testing temporal and spatial orientation, immediate and delayed verbal memory, language, attention, and praxis. The dependent variable was the total score (max. 30), corrected for age and education (Magni, Binetti, Bianchetti, Rozzini, & Trabucchi, 1996).

Alzheimer's Disease Assessment Scale-Cognitive subscale (ADAS-Cog) (Rosen, Mohs, & Davis, 1984). This tool contains 11 tasks assessing orientation, memory, language, praxis, attention, and other cognitive abilities. The dependent variable was the total score (max. 70), where higher scores indicate a more impaired cognitive functioning.

#### Language

Narrative Language Test (NLT) (Carlomagno, Vorano, Razzano, & Marini, 2013). This examines textual competence and discourse information content, assessing narrative abilities in terms of the effective communication of information. Participants are asked to describe a single figure (the “Picnic” picture in the Western Aphasia Battery) (Kertesz, 1982), and then sets of figures (two cartoon sequences) (Nicholas & Brookshire, 1993). Descriptions are recorded, transcribed verbatim, and segmented using correct information unit analysis (Nicholas & Brookshire, 1993), followed by a quantitative textual analysis (Marini, Carlomagno, Caltagirone, & Nocentini, 2005). The dependent variable was the sum of the correctly and accurately reported items.

#### Mood and behavior

Cornell Scale for Depression in Dementia (CSDD) (Alexopoulos, Abrams, Young, & Shamoian, 1988). This contains 19 items assessing signs and symptoms of major depression in individuals with dementia. Each item is rated for severity on a scale from 0 (absent) to 2 (severe). The dependent variable was the sum of the scores for the 19 items. Total scores below 6 indicate no significant depressive symptoms, those above 10 probable major depression, and those above 18 definite major depression.

Neuropsychiatric Inventory (NPI) (Cummings et al., 1994). This tool assesses 10 behavioral issues in dementia patients. The dependent variable was the total score (Frequency × Severity), which ranged from 1 to 12, with higher scores indicating more frequent and more severe behavioral problems.

#### Quality of life

Quality of Life-Alzheimer's Disease scale (QoL-AD) (Logsdon, Gibbons, McCurry, & Teri, 1999). This scale includes 13 items assessing subjective components (e.g., perceived quality of life and psychological well-being) and objective components (e.g., behavioral competence and environment) of quality of life, rated by participants on a 4-point scale from 1 (poor) to 4 (excellent). The dependent variable was the sum of all the items, where higher scores indicate a better quality of life.

### Procedure

All participants attended 20 sessions over a period of 23 weeks. Six were individual sessions for pre-test, post-test, and follow-up (about 12 weeks after intervention completion) assessments purposes, conducted by trained psychologists who did not participate in the treatment program.

During the assessment sessions, participants were administered a comprehensive battery of tests and questionnaires to assess the treatment's effectiveness (see Carbone et al., 2021 for further details).

During the other 14 sessions, participants completed the Italian adaptation (Capotosto et al., 2017) of the original CST protocol developed by Spector and colleagues (Spector et al., 2003). It consisted of 14 structured group sessions to be delivered twice a week for 7 weeks in small groups (seven-eight people), with two trained operators (one of them always a psychologist) acting as facilitators. Each session followed the same structure: i) a 10-min introduction, which included a personalized welcome, discussing a name for the group and a theme song and spatial-temporal orientation activities (discussing the day, month, year, weather and time, current affairs, and refreshments); ii) main cognitive stimulation activities, which took up 25 min, encompassing different themes across the sessions (e.g., sounds, food, categorizing objects, using money, word games), adapted, as recommended, to the participants’ cognitive abilities and divided into level A (more difficult, for people with a Mini-Mental State Examination of 19 or more) and level B (easier, for people with a Mini-Mental State Examination of 14 to 18); and iii) a 10-min conclusion, thanking everyone for attending and contributing, singing the theme song, reminding everyone of the day and time of the next session and its content, and saying goodbye. The stimulation sessions ensure that different cognitive domains (e.g.: thinking, memory, problem-solving and language skills) are appropriately engaged through a choice of activities that are adapted to the interests and abilities of the group (Gardini, 2015). However, to make sure there is continuity between the sessions, these include activities that are always the same (i.e.: the warm-up, a song, the reality orientation board at the beginning, and the closing procedures). Additionally, to augment the sense of togetherness and shared identity (Orfanos, Gibbor, Carr, & Spector, 2021) that is instrumental in fostering the supportive and non-judgmental group atmosphere crucial for facilitating positive changes in cognition and quality of life (Woods et al., 2006), the group's name and song are defined during the first session and remain the same throughout the intervention.

### Statistical analyses

Analyses were conducted using jamovi (The Jamovi Project, 2021) with the *GAMLj* module (Gallucci, 2019).

Preliminarily, any differences between the AD and VaD groups at baseline were examined for the outcomes of interest. To do this, we used independent samples *t*-tests or Mann-Whitney U tests in case of violation of distributional assumptions.

To investigate the effects of the CST-IT in the two groups across assessment sessions, we adopted a mixed-effects approach. For each outcome of interest, linear mixed models were run with dementia type (AD vs VaD), assessment session (pre-test, post-test) and their interaction as predictors, and subjects’ *id* and centers as random effects.

In cases where the linear mixed model violated distributional assumptions, a generalized mixed model with a gamma distribution was employed. Data were not available for all variables at every time point. In this case, pairwise deletion was used.

To clarify the dimension of immediate (pre- vs post-test) gains for both AD and VaD groups, Cohen's d was calculated for short-term (post-test – pre-test) and long-term (follow-up – pre-test) changes. Values were corrected using the Hedges and Olkin (1985) correction factor to avoid the small sample bias. We interpreted *d* = 0.20 as a “small” effect, *d* = 0.50 as a “medium” effect, and *d* = 0.80 or higher as a “large” effect (Cohen, 1988).

The same analyses were also run for each outcome of interest considering the follow-up assessment occurred 3 months after the treatment completion. At this assessment phase, however, participant drop-out resulted in the loss of 1 participant in the AD group and 6 participants in the VaD group, therefore, estimates in the follow-up session may be less reliable due to this imbalance in sample attrition. For this reason, these analyses have mainly descriptive/exploratory purposes. For descriptive data, mixed models, and effect sizes relative to the follow-up session, please refer to the Supplementary Material.

## Results

Table 1 shows the descriptive statistics of the outcomes of interest by assessment session and group. Results from preliminary analyses showed that there were no differences between the two groups in all the outcome measures of interest at baseline, except for neuropsychiatric symptoms; individuals with AD had a higher frequency and severity of behavioral and psychological symptoms than VaD participants (see Table 1). NPI -at baseline- was thus entered as covariate.

### Main analyses

Table 2 shows the results of the Mixed Models for the outcomes of interest only at pre-test and post-test, Table 3 provides Cohen's d for AD and VaD groups.

**Table 2:** Results from mixed-effect models for the measures of interest with dementia type (AD vs VaD), assessment session (pre-test vs post-test) and their interactions as predictors and NPI Baseline scores as covariate.

	MMSE				ADAS-cog				NLT				CSDD				NPI				QoL-AD			
Effect	*F(df)*	*B*	*t(df)*	*p*	*F(df)*	*B*	*t(df)*	*p*	*F(df)*	*B*	*z*	*p*	χ^2^*(df)*	*B*	*z*	*p*	χ^2^*(df)*	*B*	*t(df)*	*p*	*F(df)*	*B*	*t(df)*	*p*
Dementia type: VaD – AD (reference group)	<1 (1,44)	−0.11	−0.11 (44)	.92	<1 (1,44)	.41	.12 (44)	.91	<1 (1,45)	−0.39	−0.28 (45)	.78	<1 (1)	.49	.53	.60	2.15 (1)	−4.52	−1.47	.14	1.01 (1,54)	−2.29	−1.01 (54)	.32
Assessment session: Post – Pre (reference session)	**5.65** **(1,55)**	**.96**	**2.38** **(55)**	**.02**	**15.23** **(1,50)**	**−3.09**	**−3.90** **(50)**	**<0.001**	**34.00** **(1,55)**	**3.63**	**5.83** **(55)**	**<0.001**	**29.47** **(1)**	**−1.24**	**−5.43**	**<0.001**	<1 (1)	−0.17	−0.89	.37	**5.97** **(1,55)**	**1.21**	**2.44** **(55)**	**.02**
Assessment session* Dementia type	<1 (1,55)	.38	.47 (55)	.64	1.46( 1,50)	1.91	1.21 (50)	.23	<1 (1,55)	−0.67	−0.54 (55)	.59	**15.37** **(1)**	**1.79**	**3.92**	**<0.001**	<1 (1)	.17	.44	.66	**7.00** **(1,55)**	**2.62**	**2.65** **(55)**	**.01**
NPI Baseline	<1 (1,52)	.04	.92 (52)	.36	<1 (1,49)	−0.05	−0.39 (49)	.70	**6.12** **(1,53)**	**.13**	**2.47** **(53)**	**.02**	**24.49** **(1)**	**.19**	**4.95**	**<0.001**	–	**–**	**–**	**–**	**5.05** **(1,52)**	**−0.18**	**−2.25** **(52)**	**.03**

**Note**: *MMSE = Mini-Mental State Examination; ADAS-Cog = Alzheimer's Disease Assessment Scale – Cognitive Subscale; NLT = Narrative Language Test; CSDD = Cornell Scale for Depression in Dementia; NPI = Neuropsychiatric Inventory; QoL-AD = Quality of Life in Alzheimer's Disease Scale; F = fixed effect test for linear mixed models, χ^2^ = fixed effect test for generalized mixed models with gamma distribution. Significant results in bold*.

**Table 3:** Effect sizes (Cohen's d) for short-term (pre-test vs post-test) CST-IT benefits in AD and VaD groups.

	AD	VaD
MMSE	0.20	0.30
ADAS-Cog	0.31	0.23
NLT	0.62	0.67
CSDD	0.74	0.38
NPI	0.41	0.14
QoL-AD	−0.01	0.30

**Note:***AD* = *Alzheimer's Disease; VaD* = *Vascular Dementia; MMSE* = *Mini-Mental State Examination; ADAS-Cog* = *Alzheimer's Disease Assessment Scale – Cognitive Subscale; NLT* = *Narrative Language Test; CSDD* = *Cornell Scale for Depression in Dementia; NPI* = *Neuropsychiatric Inventory; QoL-AD* = *Quality of Life in Alzheimer's Disease Scale. A positive Cohen's d indicates an improvement in the outcome, while a negative one indicates a worsening in the outcome.*

Regarding cognitive outcomes, a significant main effect of assessment session was observed for the MMSE regardless of dementia type, *F*(1,55) = 5.65, *p* = .02. Both groups showed improvements in global cognitive functioning at post-test*.* Neither dementia type nor assessment session * dementia type interaction were significant (see Table 2).

The same result was found for the ADAS-Cog: a significant main effect of assessment session was observed, *F*(1,50) = 15.23, *p* < 0.001, with both AD and VaD participants showing a higher general cognitive functioning at post-test. Neither dementia type nor assessment session * dementia type interaction were significant (see Table 2).

As for the NLT, results again showed a significant main effect of assessment session, regardless of dementia type, *F*(1,55) = 34.00, *p* < .001. Improvements were observed in both AD and VaD at post-test. Neither dementia type nor assessment session * dementia type interaction were significant (see Table 2). A significant effect of the covariate NPI Baseline was found, *F*(1,53) = 6.12, *p* = .02, suggesting that frequency and severity of neuropsychiatric symptoms at baseline contributed to explain the observed variance in narrative informativeness (see Table 2).

Concerning mood, we employed a generalized mixed model due to the violation of distributional assumptions. There was a significant main effect of assessment session, *χ^2^*(1) = 29.47, *p* < .001, and a significant assessment session * dementia type interaction, *χ^2^*(1) = 15.37, *p* < .001, for the CSDD. There was a decrease in depressive symptoms in both groups at post-test, with AD showing a greater decrease compared to VaD. The main effect of dementia type was not significant (see Table 2). However, the main effect was conditional to frequency and severity of neuropsychiatric symptoms at baseline *χ^2^*(1) = 24.49, *p* < .001, as it varied across degrees of behavioral and mood disruptions (see Table 2).

We employed a generalized mixed model for neuropsychiatric symptoms, as well. There was no significant main effects nor interaction on the NPI outcome (see Table 2).

In quality of life, a significant main effect of assessment session, *F*(1,55) = 5.97, *p* = .02, and assessment session * dementia type interaction, *F*(1,55) = 7.00, *p* = .01, emerged. There was no change in AD at post-test, while in VaD there was an improvement. The main effect of dementia type was not significant (see Table 2). Again, a significant effect of the NPI Baseline was found, *F*(1,52) = 5.05, *p* = .03), confirming the negative relationship between behavioral and psychological symptoms and QoL (see Table 2).

Regarding effect sizes, for global cognitive functioning Cohen's d were small for MMSE and ADAS-Cog for both groups (see Table 3). As for NLT, effect sizes were medium for both groups. Regarding depressive symptoms, Cohen's d was medium for AD and small for VaD. For neuropsychiatric symptoms, effect sizes were small for AD and negligible for VaD, whereas for QoL effect sizes were negligible for AD and small for VaD.

### Exploratory analyses

When considering the follow-up assessment (see Supplementary Materials for details), both groups demonstrated an improvement in global cognitive functioning as measured by the MMSE, which remained stable at follow-up, *F*(2103) = 3.26, *p* = .04. Differently, AD and VaD participants improved at post-test in the ADAS-Cog but returned to initial levels at follow-up, *F*(2,93) = 7.64, *p* = .005 (see Supplemental Tables 2–3).

For the NLT, a significant main effect of assessment session, *F*(2104) = 15.94, *p* < .001, and an assessment session * dementia type interaction, *F*(2104) = 4.68, *p* = .01, emerged. At follow-up, improvements in language remained stable only in VaD, while there was a return to baseline levels in AD. A significant main effect of the assessment session (*χ^2^*(2) = 24.40, *p* < .001) and a significant assessment session * dementia type interaction (*χ^2^*(2) = 11.43, *p* = .003) were found for the CSDD. At follow-up, the decrease in depressive symptoms remained stable only in AD, while there was a return to baseline levels for VaD. In quality of life, a significant assessment session * dementia type interaction emerged, *F*(2103) = 3.54, *p* = .03. There was no change in AD across the assessment sessions, while in VaD the observed improvement at post-test remained stable at follow-up.

Concerning effect sizes for long-term benefits, Cohen's d in global cognitive functioning (i.e., MMSE and ADAS-Cog), were overall negligible. As for NLT, effect sizes were large for VaD and negligible for AD. Regarding depressive symptoms, Cohen's d were small for AD and negligible for VaD. For neuropsychiatric symptoms and QoL, effect sizes were overall negligible (see Supplemental Table 4).

## Discussion

Further investigation is necessary regarding the efficacy of cognitive stimulation in dementia subtypes other than AD (Woods et al., 2023), while also delving into the mechanisms of change induced by CST and their associations with the pathological processes linked to various forms of dementia. Hence, the present study aimed at conducting a comparative analysis of the CST-IT program effects on distinct subgroups of patients affected by AD and VaD.

Extending previous research, we demonstrated a positive impact of the CST-IT intervention on measures of global cognition, with a comparable effect size in both subgroups after intervention completion. Although analyses considering also the follow-up assessment were only exploratory in nature, when cognitive functioning was measured using the MMSE such improvement seemed to remain stable from pre-test to the 3-months follow-up in both the AD and the VaD sample, while for the ADAS-Cog a non-significant decrease in effect sizes was observable in both groups. The reported immediate effect is perfectly in line with previous meta-analytic evidence (Woods et al., 2023) demonstrating a small, though significative, improvement in cognition after intervention completion (around 2.66 points on the ADAS-Cog and 2.16 on the MMSE), while scanty evidence exists on maintenance of such benefits (Woods et al., 2023). Indeed, the above-mentioned meta-analysis (Woods et al., 2023) demonstrated that, compared to patients not receiving any cognitive interventions, larger long-term effects (at three months from the treatment completion) were observable in the MMSE compared to the ADAS-Cog, although results were moderately inconsistent, with a high level of imprecision, thereby casting doubt on whether any improvement is maintained in the long-term.

It is worth mentioning that a recent network meta-analysis (Sun, Zhang, & Wang, 2022) established that maintenance CST, which was developed as a further CST protocol with additional 24 weekly sessions (Aguirre et al., 2013), was the most effective CST setting (compared to the 7-week CST program, and to a home-based, one-to-one version of CST facilitated by informal/formal caregivers). Such a result thus plausibly suggests that an extended duration of the intervention may lead to more enduring benefits for individuals with cognitive decline, irrespective of the specific underlying pathological condition. However, no evidence subsists for now that a greater number of sessions led to greater effect sizes (Woods et al., 2023), while the effect of total exposure on maintenance of benefits has never been explored. These issues are therefore worth investigating.

Considering specific cognitive abilities, i.e. informativeness in referential narratives, we found that both groups improved immediately after intervention, as also envisaged by the medium effect sizes displayed by both groups. A significant effect of diagnosis emerged only when long-term benefits were concerned. As a matter of fact, a significant difference between effect sizes for long-term changes in the NLT was observable between the two groups. VaD patients seemed to maintain a better capability in describing the core of complex pictures, whereas AD individuals demonstrated a substantial decline in informativeness in comparison to their post-intervention levels. Indeed, narrative language and communication are among the cognitive domains that appear to be most influenced by CST (Desai et al., 2024; Sun et al., 2022; Woods et al., 2023), possibly because of the language-based nature of the intervention that would enhance neural pathways responsible for processing of syntax, potentially also aiding verbal recall (Hall, Orrell, Stott, & Spector, 2013). The interpersonal aspects of CST, the positive reinforcement of thinking, questioning, and expressing opinions during the program, and the generation of new semantic links through categorization (Spector & Orrell, 2010) would thus sustain, in dementia affected persons (regardless of the underlying pathological process), the neuronal networks responsible for informative communication, eventually promoting the functioning of alternative pathways (Hall et al., 2013). However, in VaD patients the neuropsychological mechanism of change might be slightly different, and based on the effect that CST exerts on other cognitive abilities. Indeed, we previously demonstrated a trend toward significance (which might have reached statistical significance in a larger sample) for the intervention effect on short-term/working memory in patients clinically diagnosed with VaD (Piras et al., 2017). Since story production is sustained by neural networks supporting the integration, maintenance and manipulation of multimodal information (Mar, 2004), it can be suggested that the enduring improvement in narrative abilities in VaD patients was promoted by CST-IT-induced changes in working-memory processes. Actually, it has been advocated that a potential mechanism for the effects of CST could be via improving cognitive flexibility in language and working memory (Desai et al., 2024), and the present findings might corroborate such hypothesis. Alternatively, considering that VaD patients do not exhibit impairments in the semantic and pragmatic levels of language processing as seen in AD and other types of dementia (Karantzoulis & Galvin, 2011), it can be suggested that the CST-IT program enhanced the social confidence of individuals affected by vascular cognitive deterioration, allowing them to practice and experience communicative successes in a supportive environment (Woods et al., 2023), thereby endurably improving their ability to convey information efficiently and effectively. Intriguingly, the reported effect on communicative informativeness was conditional to frequency and severity of neuropsychiatric symptoms at baseline (which were expectably, more prevalent in the AD sample), as the covariate significantly contributed to explain the observed variance in the number of correctly reported elements depicting the core of complex pictures and animated strips. Although is still unclear whether neuropsychiatric symptoms precede or are a consequence of cognitive impairment, or whether there is a bidirectional or more complex relationship between them (Sabates et al., 2024), the present findings suggest that the intervention effect on language productivity was not constant across different levels of frequency or severity of neuropsychiatric symptoms.

The same relationship was observable for depressive symptoms, where the significant decrease in symptomatology at intervention completion, with a significant interaction of diagnosis such that AD patients benefitted more from the program, was conditional to frequency and severity of baseline neuropsychiatric symptoms. The CST positive impact on affective symptoms is a robust finding (Desai et al., 2024; Woods et al., 2023), contributing to justify the tradeoff between the extensiveness of the intervention and the small impact on cognition, as additional changes in mood, behavior and quality of life are consistently observed after the CST program (Woods et al., 2023). While in our study no effect was detected on neuropsychiatric symptoms, depressive symptoms were significantly reduced immediately after the program, with a more evident change in AD (approximately 1 standard deviation above that of VaD individuals, when groups’ effect sizes were compared). This improvement was sustained throughout the post-intervention period up to the 3-months follow-up, albeit with a decrease in efficacy observed particularly in VaD.

Such interplay between CST-IT impact and the underlying pathophysiology can be elucidated considering that vascular disorders impair the anatomical substrates involved in the onset of depressive symptoms. Moreover, the manifestation of vascular depression is consistently linked to the compromised functionality of frontostriatal neural circuits along with their connections to limbic and hippocampal regions, leading to the depression-executive dysfunction profile characteristic of vascular cognitive impairment and dementia (Alexopoulos, 2003). While CST has demonstrated efficacy in addressing certain dysexecutive symptoms (Desai et al., 2024) also in VaD patients (Piras et al., 2017), its therapeutic benefits could be constrained when targeting the management of vascular depressive symptomatology, which is characterized by pharmacological resistance and unstable treatment response due to subcortical lesions disrupting the connectivity between dorsal cortical structures and ventral limbic regions (Alexopoulos, Kiosses, Choi, Murphy, & Lim, 2002). Alternatively, the de-stigmatizing nature of CST (Hall et al., 2013), its person-centered approach and the provision of a supportive/non-threatening group environment and social comfort may have a stronger impact on depressive symptoms in AD patients, who can experience social withdrawal, low self-esteem and hopelessness due to internalized negative stereotypes associated with the disease (Rosin, Blasco, Pilozzi, Yang, & Huang, 2020).

A further differential impact of the CST-IT program was observable on self-reported quality of life, which improved (and remained stable three months after the program completion) in VaD patients, while no significant change was reported in AD. This finding is at variance with the robust effect (albeit small) (Desai et al., 2024; Woods et al., 2023) usually observed after the CST program in quality of life, and contrasts with our own previous findings of a null CST-IT impact on self-reported quality of life in VaD. However, the observed change significantly varied according to frequency and severity of neuropsychiatric symptoms at baseline, thus possibly explaining the unexpected result. Indeed, although improvements in quality of life are mediated by positive changes in cognition (Woods et al., 2006), therefore explaining the observed interconnection between cognitive/social stimulation and enhanced emotional, social and physical well-being, more frequent and severe neuropsychiatric symptoms are associated with lower quality of life (Appelhof et al., 2017). Given the observed differences between AD and VaD patients regarding challenging behaviors in the current sample, as well as the inverse relationship noted between baseline NPI scores and the primary CST-IT impact on quality of life, it is conceivable that individuals with fewer behavioral disturbances (specifically VaD patients) could derive a greater benefit from the intervention, thereby manifesting a substantial improvement in their overall life satisfaction. Additionally, as previously suggested (Woods et al., 2006), self-reported quality of life is significantly associated with improvements in communicative abilities, and the enduring significant change in VaD patients’ narrative efficiency may have contributed to the observed differential change in perceived emotional, social and physical quality of life.

## Conclusions

Rather than assuming that an intervention can have universal advantages, and acknowledging the possible differential impact of the CST program on individuals with dementia (Woods et al., 2023), we intended to explore the potential variations in CST-IT-related changes based on the specific subtype of dementia diagnosis. We found that the intervention determined comparable clinically significant improvements in general cognition and communicative abilities, thus demonstrating that the program can prevent months of decline in people living with dementia (Woods et al., 2023), regardless of the underlying pathological process. A significant interaction with dementia subtype was however observable, immediately following intervention completion, in other significant outcomes like depressive symptoms (with a greater improvement in AD patients and a yet appreciable change in VaD) and self-reported quality of life (where no significant effect was observable in AD, while VaD showed a small improvement). Nevertheless, the reported effects were conditional to diagnosis-related variances in neuropsychiatric symptoms (with AD patients expectably experiencing more frequent and severe behavioral and mood disruptions), which also impacted the described changes in narrative abilities. Likewise, although purely exploratory, the investigation of long-term effects revealed that maintenance of gains varied according to dementia diagnosis as improvements in narrative informativeness were maintained in VaD, while in AD communicative abilities returned to baseline levels. Contrariwise, the post-intervention change in depressive symptoms in AD was still observable three months after intervention completion, while in VaD became negligible.

Different mechanisms of neuropsychological change after CST-IT can be hypothesized for different forms of dementia. It is thus necessary to consider the interconnected correlation between neuropsychiatric manifestations and cognitive characteristics in subtypes of dementia, as this could significantly influence the effectiveness of the CST program, particularly with respect to crucial outcome measures such as mood and quality of life. These findings should serve as a stimulus for researchers to conduct more extensive investigations into the simultaneous occurrence of neuropsychiatric and cognitive symptoms across various types of dementia, as well as to explore therapeutic approaches that tackle both categories of symptoms concurrently, while also considering the distinct cognitive-behavioral profile of neurodegenerative disorders.

A few limitations of the present study should, however, be acknowledged before concluding remarks. First, the sample size is limited, although the population was drawn from a previous single-blind, multicenter, controlled clinical trial (Carbone et al., 2021) involving a much larger group (123 patients with mild-to-moderate dementia and 102 comparators). As we did not intend to establish CST-IT efficacy (which was demonstrated in the previous study), but rather explore potential variations according to diagnosis in immediate and long-term gains derived from the program, we think that the present analyses are sufficiently powered. Indeed, a post-hoc power analysis (F test for a repeated measures ANOVA, within and between interaction, α=0.05, *N* = 48) considering the difference between Hedge's g of two treatment groups in pre-post research designs for the language measure (our strongest result considering the differential effect of the CST-IT in the two groups, d_coor_ = 0.65) demonstrated that the eventuality of a Type II error was remote (Power (1-ß error probability) =0.99). However, owing to the different attrition rates, findings on long-term effects should be considered purely exploratory. Additionally, we were not able to compute the potential impact of losses at post-intervention and follow-up on the reported CST-IT effects (only per-protocol measures are included) and cannot disclose the reasons for patients not undergoing the scheduled assessments. Nevertheless, given our objective to examine the potential impact of dementia type on improvements resulting from the intervention, and their correlation with variations in behavioral and cognitive symptoms related to diagnosis, we believe that the disparity in drop-out rates did not significantly affect the reported findings.

Even that VaD represents a heterogenic entity from the point of view of histopathological substrates, up-to-date there are no specific interventions regarding cognitive rehabilitation/stimulation (Balea et al., 2018). Although we demonstrated that different neuropsychological mechanisms of change possibly subtend the observed improvements after CST-IT in this subgroup, we can affirm that the program is effective in determining significant changes in general cognition (around the minimal pre-post difference in ADAS-Cog and MMSE considered as clinically relevant (Schrag & Schott, 2012), communicative abilities and quality of life, thus substantiating its effectiveness also in patients with a predominant vascular contribution to cognitive deterioration. Currently, our comprehension of the cognitive and behavioral characteristics of various other neurodegenerative conditions, along with the possible beneficial impact of psychosocial interventions, is less advanced compared to that of AD (Levy & Chelune, 2007). Thus, the observation that CST seems to be similarly efficacious in the most prevalent types of dementia when general cognitive functioning is concerned, but also holds the potential to provide nuanced benefits in outcomes targeting specific cognitive domains, mood and quality of life depending on the dementia etiology, carries significant consequences for theoretical frameworks and practical applications in the clinical setting.

## Data statement

The data that support the findings of this study are available from the corresponding author upon reasonable request.

## Declaration of competing interest

The authors declare that they have no known competing financial interests or personal relationships that could have appeared to influence the work reported in this paper.

## Appendix Supplementary materials

Image, application 1
