# Supplementary material for: Does Cognitive Stimulation Therapy show similar efficacy in individuals with mild-to-moderate dementia from varying etiologies? An examination comparing its effectiveness in Alzheimer's disease and vascular dementia
Source: Int J Clin Health Psychol. 2024 Oct 18;24(4):100510. doi: 10.1016/j.ijchp.2024.100510 (PMC11513487; doi:10.1016/j.ijchp.2024.100510)
Supplement: Supplementary file 1 [file mmc1.docx]

**Supplementary Materials**

**Does Cognitive Stimulation Therapy show similar efficacy in individuals with mild-to-moderate dementia from varying etiologies? An examination comparing its effectiveness in Alzheimer's disease and vascular dementia**

During the follow-up phase, participant drop-out resulted in the loss of 1 subject in the AD group and 6 subjects in the VaD group. In the manuscript, we did not include the results related to the follow-up data (i.e., long-term changes). We decided to report this data here in the Supplementary Materials.

Table S1 shows the descriptive statistics of the outcomes of interest by assessment session (pre-test, post-test, follow-up) and group (AD vs VaD). Tables S2-S3 show the results of the Mixed Models for the outcomes of interest. Table 4 provides Cohen’s d for short- and long-term changes by group.

Regarding global cognitive functioning, a significant main effect of assessment session was observed for both the MMSE regardless of dementia type, *F*(2,103) = 3.26, *p* = .04. Both groups showed improvements in the global cognitive functioning at post-test that remained stable at follow-up*.* Neither dementia type nor assessment session * dementia type interaction were significant.

For the ADAS-Cog, a significant main effect of assessment session emerged, *F*(2,93) = 7.64, *p* = .005. AD and VaD participants improved at post-test but returned to the initial levels at follow-up. Neither dementia type nor assessment session * dementia type interaction were significant.

As for the NLT, results showed a significant main effect of assessment session, *F*(2,104) = 15.94, *p* < .001, and assessment session * dementia type interaction, *F*(2,104) = 4.68, *p* = .01. Improvements were observed in both AD and VaD at post-test, but at follow-up this improvement remained stable only in VaD, while there was a return to baseline levels in AD. The main effect of dementia type was not significant. A significant effect of the covariate NPI Baseline was found, *F*(1,53) = 5.48, *p* = .02, indicating that frequency and severity of neuropsychiatric symptoms at baseline contributed to explain the observed variance in narrative informativeness.

Concerning mood and behavior, we employed a generalized mixed model due to the violation of distributional assumptions. A significant main effect of the assessment session, *χ^2^*(2) = 24.40, *p* < .001, and a significant assessment session * dementia type interaction, *χ^2^*(2) = 11.43, *p* = .003, were found for the CSDD. There was a decrease in depressive symptoms in both groups at post-test, with AD showing a greater decrease compared to VaD; while in the follow-up this improvement remained stable only in AD, while there was a return to baseline levels for VaD. The main effect of dementia type was not significant. There was a significant effect of the covariate NPI Baseline, *χ^2^*(1) = 21.81, *p* < .001, indicating that the effect was conditional to frequency and severity of neuropsychiatric symptoms at baseline, as it varied across degrees of behavioral and mood disruptions.

We employed a generalized mixed model for neuropsychiatric symptoms, as well. There was no significant main effects nor interaction on the NPI outcome.

In quality of life, a significant assessment session * dementia type interaction emerged, *F*(2,103) = 3.54, *p* = .03. There was no change in AD across the assessment sessions, while in VaD there was an improvement at post-test that remained stable at follow-up. Neither dementia type nor assessment session were significant. The NPI Baseline covariate resulted significant, *F*(1,52) = 4.55, *p* = .04, confirming the negative relationship between behavioral and psychological symptoms and QoL.

Regarding effect sizes, for global cognitive functioning (i.e., MMSE and ADAS-Cog) Cohen’s d were overall small at short-term and negligible at long-term for these outcomes. As for NLT, effect sizes were medium at short-term for both groups and large for VaD and negligible for AD at long-term. Regarding depressive symptoms, Cohen’s d was medium for AD and small for VaD at short-term and small for AD and negligible for VaD at long-term. For neuropsychiatric symptoms, effect sizes were small at post-test and then negligible at follow-up for AD, but negligible for VaD at all assessments session. Finally, for QoL effect sizes were negligible for AD and small for VaD at post-test and negligible at follow-up.

**Table S1**. Descriptive statistics of the measures of interest by group (AD and VaD) and assessment session (pre-test, post-test and follow-up).

|  | AD  (N = 30; 19 females) | | | | | | | | | VaD  (N = 27; 22 females) | | | | | | | | |
| --- | --- | --- | --- | --- | --- | --- | --- | --- | --- | --- | --- | --- | --- | --- | --- | --- | --- | --- |
|  | *Pre-test* | | | *Post-test* | | | *Follow-up* | | | *Pre-test* | | | *Post-test* | | | *Follow-up* | | |
|  | *N* | *M* | *SD* | *N* | *M* | *SD* | *N* | *M* | *SD* | *N* | *M* | *SD* | *N* | *M* | *SD* | *N* | *M* | *SD* |
| MMSE | 30 | 19.64 | 3.59 | 30 | 20.41 | 4.08 | 29 | 20.08 | 4.15 | 27 | 19.63 | 3.43 | 27 | 20.78 | 4.46 | 21 | 20.95 | 4.70 |
| ADAS-Cog | 26 | 28.99 | 12.79 | 26 | 24.94 | 13.63 | 25 | 27.42 | 14.07 | 26 | 26.84 | 8.97 | 26 | 24.71 | 9.17 | 20 | 25.64 | 8.96 |
| NLT | 30 | 12.17 | 4.91 | 30 | 16.13 | 7.53 | 29 | 12.52 | 5.25 | 27 | 10.07 | 4.50 | 27 | 13.37 | 5.20 | 21 | 13.48 | 3.79 |
| CSDD | 30 | 7.40 | 5.12 | 30 | 4.17 | 3.44 | 29 | 5.48 | 4.52 | 27 | 5.41 | 4.99 | 27 | 3.70 | 3.90 | 21 | 5.24 | 4.46 |
| NPI | 30 | 16.30 | 13.05 | 30 | 11.37 | 11 | 29 | 18.66 | 15.44 | 27 | 7.81 | 11.76 | 27 | 6.41 | 8.04 | 21 | 8.62 | 11.29 |
| QoL-AD | 30 | 31.73 | 8.51 | 30 | 31.63 | 7.51 | 29 | 31.21 | 7.59 | 27 | 27.63 | 8.97 | 27 | 30.15 | 7.54 | 21 | 29.86 | 6.69 |

**Note**: *AD = Alzheimer’s Disease; VaD = Vascular Dementia; CDR = Clinical Dementia Rating; MMSE = Mini-Mental State Examination; ADAS-Cog = Alzheimer’s Disease Assessment Scale – Cognitive Subscale; CSDD = Cornell Scale for Depression in Dementia; NPI = Neuropsychiatric Inventory; QoL-AD = Quality of Life in Alzheimer’s Disease Scale.*

**Table S2.** Results from mixed-effect models for the measures of interest with dementia type (AD vs VaD), assessment session (pre-test vs post-test vs follow-up) and their interactions as predictors and NPI Baseline scores as covariate. Fixed effect omnibus test.

|  | **MMSE** | | **ADAS-Cog** | | **NLT** | | **CSDD** | | **NPI** | | **QoL-AD** | |
| --- | --- | --- | --- | --- | --- | --- | --- | --- | --- | --- | --- | --- |
|  | **F*(df)*** | ***p*** | **F*(df)*** | ***p*** | **F*(df)*** | ***p*** | **χ^2^*(df)*** | ***p*** | **χ^2^*(df)*** | ***p*** | **F*(df)*** | ***p*** |
| Dementia type | < 1 | .83 | < 1 | .97 | < 1 | .74 | .31(1) | .58 | 1.89(1) | .17 | < 1 | .34 |
| Assessment session | **3.26(2,103)** | **.04*** | **7.64(2,93)** | **.005**** | **15.94(2,104)** | **<.001***** | **24.40(2)** | **<.001***** | 3.60(2) | .17 | 2.76(2,103) | .07 |
| Assessment session ✻ Dementia type | < 1 | .84 | < 1 | .49 | **4.68(2,104)** | **.01*** | **11.43(2)** | **.003**** | .27(2) | .87 | **3.54(2,103)** | **.03*** |
| NPI Baseline | 1.24(1,51) | .27 | < 1 | .62 | **5.48(1,53)** | **.02*** | **21.81(1)** | **<.001***** | \ | \ | **4.55(1,52)** | **.04*** |

**Note**: *MMSE = Mini-Mental State Examination; ADAS-Cog = Alzheimer’s Disease Assessment Scale – Cognitive Subscale; NLT = Narrative Language Test; CSDD = Cornell Scale for Depression in Dementia; NPI = Neuropsychiatric Inventory; QoL-AD = Quality of Life in Alzheimer’s Disease Scale; F = fixed effect test for linear mixed models, χ^2^ = fixed effect test for generalized mixed models with gamma distribution. Significant results in bold.*

**Table S3**. Results from mixed-effect models for the measures of interest with dementia type (AD vs VaD), assessment session (pre-test vs post-test vs follow-up) and their interactions as predictors and NPI Baseline scores as covariate. Fixed effects parameter estimates.

|  | **MMSE** | | | **ADAS-Cog** | | | **NLT** | | | **CSDD** | | | **NPI** | | | **QoL-AD** | | |
| --- | --- | --- | --- | --- | --- | --- | --- | --- | --- | --- | --- | --- | --- | --- | --- | --- | --- | --- |
| **Effect** | ***B*** | ***t(df)*** | ***p*** | ***B*** | ***t(df)*** | ***p*** | ***B*** | ***t(df)*** | ***p*** | ***B*** | ***z*** | ***p*** | ***B*** | ***z*** | ***p*** | ***B*** | ***t(df)*** | ***p*** |
| Dementia type: VaD – AD (reference group) | .24 | .22(43) | .83 | -.37 | -.11(43) | .92 | .42 | .34(48) | .74 | .52 | .56 | .58 | -4.52 | -1.37 | .17 | -2.11 | -.97(54) | .34 |
| Assessment session: Post – pre (reference session) | **.96** | **2.51(103)** | **.01** | **-3.09** | **-3.85(93)** | **<.001** | **3.63** | **5.64(103)** | **<.001** | **-1.30** | **-4.93** | **<.001** | -.16 | -.68 | .50 | **1.21** | **2.34(103)** | **.02** |
| Assessment session: Follow-up - pre | **.64** | **1.59(104)** | **.01** | -1.02 | -1.19(94) | .24 | **1.90** | **2.81(105)** | **.006** | **-1.02** | **-3.25** | **.001** | .91 | 1.59 | .11 | .51 | .92(103) | .36 |
| Assessment session: Post - pre*Dementia type: VaD - AD | .38 | .50(103) | .62 | 1.91 | 1.19(93) | .24 | -.67 | -.52(103) | .60 | **1.63** | **3.10** | **.002** | .17 | .38 | .71 | **2.62** | **2.54(103)** | **.01** |
| Assessment session: Follow-up - pre*Dementia type: VaD - AD | .38 | .51(103) | .61 | 1.26 | .74(94) | .46 | **3.27** | **2.41(105)** | **.02** | **1.97** | **3.15** | **.002** | .49 | .43 | .67 | 2.09 | 1.91(103) | .06 |
| NPI Baseline | .05 | .22(43) | .27 | -.06 | -.49(48) | .62 | **.11** | **2.34(53)** | **.02** | **.18** | **4.67** | **<.001** | \ | \ | \ | **-.17** | **-2.13(52)** | **.04** |

**Note:** *AD = Alzheimer’s Disease; VaD = Vascular Dementia; MMSE = Mini-Mental State Examination; ADAS-Cog = Alzheimer’s Disease Assessment Scale – Cognitive Subscale; NLT = Narrative Language Test; CSDD = Cornell Scale for Depression in Dementia; NPI = Neuropsychiatric Inventory; QoL-AD = Quality of Life in Alzheimer’s Disease Scale.*

**Table S4**. Effect sizes (Cohen’s d) for short-term (pre-test vs post-test) and long-term changes (pre-test vs follow-up) due to CST in AD and VaD groups.

|  | **AD** | | **VaD** | |
| --- | --- | --- | --- | --- |
|  | **Short-term** | **Long-term** | **Short-term** | **Long-term** |
| **MMSE** | 0.20 | 0.07 | 0.30 | 0.17 |
| **ADAS-Cog** | 0.30 | 0.12 | 0.23 | 0.02 |
| **NLT** | 0.61 | 0.07 | 0.66 | 0.81 |
| **CSDD** | 0.73 | 0.41 | 0.38 | 0.19 |
| **NPI** | 0.40 | -0.13 | 0.14 | -0.04 |
| **QoL-AD** | -0.01 | -0.07 | 0.30 | 0.19 |

**Note:** *AD = Alzheimer’s Disease; VaD = Vascular Dementia; MMSE = Mini-Mental State Examination; ADAS-Cog = Alzheimer’s Disease Assessment Scale – Cognitive Subscale; NLT = Narrative Language Test; CSDD = Cornell Scale for Depression in Dementia; NPI = Neuropsychiatric Inventory; QoL-AD = Quality of Life in Alzheimer’s Disease Scale. A positive Cohen's d indicates an improvement in the outcome, while a negative one indicates a worsening in the outcome.*
